# Crystallographic origin of pressure-induced bond order in the frustrated spinel CuIr_2_S_4_

**DOI:** 10.1038/s42004-026-02240-y

**Published:** 2026-10-26

**Authors:** Masatoshi Emi, Tsubasa Ohashi, Keita Kojima, Koudai Sugimoto, Kazuyuki Matsubayashi, Kenta Oka, Hirokazu Kadobayashi, Saori Kawaguchi-Imada, Daigo Ito, Taisei Kubo, Kenta Hashimoto, Hitoshi Kawaji, Hiroyuki S. Suzuki, Shoichi Nagata, Naoyuki Katayama

**Affiliations:** 1 Department of Applied Physics, Nagoya University, Nagoya, Japan; 2 Graduate School of Environmental, Life, Natural Science and Technology, Okayama University, Okayama, Japan; 3 The Institute for Solid State Physics, The University of Tokyo, Kashiwa, Japan; 4 Department of Physics, Keio University, Yokohama, Japan; 5 Department of Physics, Kitasato University, Sagamihara City, Japan; 6 Department of Engineering Science, The University of Electro-Communications, Chofu, Tokyo, Japan; 7 Japan Synchrotron Radiation Research Institute (JASRI), SPring-8, Hyogo, Japan; 8 Office of Institutional Advancement and Communications, Kyoto University, Kyoto, Japan; 9 Materials and Structures Laboratory, Institute of Science Tokyo, Yokohama, Japan; 10 Department of Materials Science and Engineering, Muroran Institute of Technology, Hokkaido, Japan

**Keywords:** Electronic properties and materials, Chemical bonding, Electronic materials

## Abstract

CuIr_2_S_4_ is a frustrated pyrochlore spinel in which pressure reshapes competing charge- and bond-ordered states. Here we determine the crystallographic nature of its pressure-induced ordered phases up to about 25 GPa by high-pressure synchrotron powder X-ray diffraction combined with first-principles-guided structural analysis. Pressure first stabilizes the ambient-pressure low-temperature ordered state to room temperature and then induces a distinct high-pressure phase. Using single-phase datasets collected at 19.8 and 25.7 GPa, we perform full-atom refinements that consistently establish the Phase-IV symmetry and Ir–Ir short-bond topology. The resulting Phase IV pattern differs from Phase II and is not captured by a simple orbitally induced Peierls picture. Instead, it is consistent with an Anderson-type local tetrahedron rule on the pyrochlore lattice. Total-energy calculations reproduce a pressure-induced reversal in the relative stability of the Phase-II- and Phase-IV-type structural models.

## Introduction

In pyrochlore lattices, strong competition among ordered states arises from geometric frustration inherent to the corner-sharing tetrahedral network^1,2^. Consequently, even when an apparently stable ordered state is realized at low temperatures, external stimuli can reconstruct the free-energy landscape and stabilize alternative ordered states. For example, in MnV_2_O_4_, strong spin–lattice coupling has been reported to cause structural changes upon application of a magnetic field^3–5^. In CuIr_2_S_4_, multiple structural phase transitions have been reported under pressure, with metallization and superconductivity emerging at ultrahigh pressures^6,7^. While the diverse electronic phases induced by external fields make pyrochlore systems an attractive research arena, their structural determination often presents substantial technical challenges. In particular, pressure-induced ordered phases commonly exhibit pronounced symmetry lowering and phase coexistence, requiring analysis strategies tailored to the material and data quality to establish reliable crystallographic models.

In CuIr_2_S_4_, our current research target, a low-temperature ordered phase accompanied by charge ordering and Ir–Ir dimer formation has been reported^8–15^. The crystal structure of this phase was recently refined by Ohashi et al. using synchrotron single-crystal X-ray diffraction, providing updated crystallographic parameters^15^. The resulting bond disproportionation (short-bond arrangement) is consistent with the orbitally induced Peierls scenario discussed by Khomskii and Mizokawa^16^. On the other hand, within an idealized ionic picture, the charge arrangement in this low-temperature phase does not strictly satisfy an Anderson-type local tetrahedron rule on the pyrochlore lattice, suggesting that competing charge/bond configurations may be close in energy^17^. Under pressure, the low-temperature phase is stabilized to higher temperatures and becomes observable even at room temperature. With further compression, multiple high-pressure phases, including coexistence regimes, have been reported at room temperature. However, unresolved issues remain regarding the crystallographic nature—particularly the symmetry and short-bond (dimer) pattern—of the ordered phase appearing in the intermediate-pressure region. Because pressure-induced ordered states in frustrated spinels often involve low symmetry, enlarged unit cells, and phase coexistence, their structural determination from DAC powder diffraction data is generally nontrivial. In the present study, we use first-principles calculations not as a substitute for diffraction analysis, but as a physically motivated way to construct an initial structural model for the high-pressure phase.

This paper focuses on the structural evolution through the Phase-II + IV coexistence regime and on the determination of the Phase-IV crystal structure up to ~25 GPa. A two-phase coexistence region exists between Phases II and IV, and the primary objective of this study is to determine the crystal structure of Phase IV. Starting from a DFT-relaxed model, we analyze single-phase diffraction datasets collected in the high-pressure regime and show that the atomic coordinates of all constituent elements can be refined stably, consistently yielding the same Phase-IV symmetry and Ir–Ir short-bond topology. The resulting Phase IV structure exhibits a charge/bond pattern distinct from that of Phase II and cannot be fully captured by a simple orbitally induced Peierls picture. Instead, the pattern is consistent with an Anderson-type local tetrahedron rule on the pyrochlore lattice^17–19^. Total-energy calculations reproduce a pressure-induced reversal in the relative stability of the Phase-II- and Phase-IV-type structural models. Together, these findings provide a direct crystallographic basis for pressure-driven order selection in a frustrated pyrochlore-lattice system.

## Results and discussion

### XRD measurements under high pressure

**Fig. 1:**
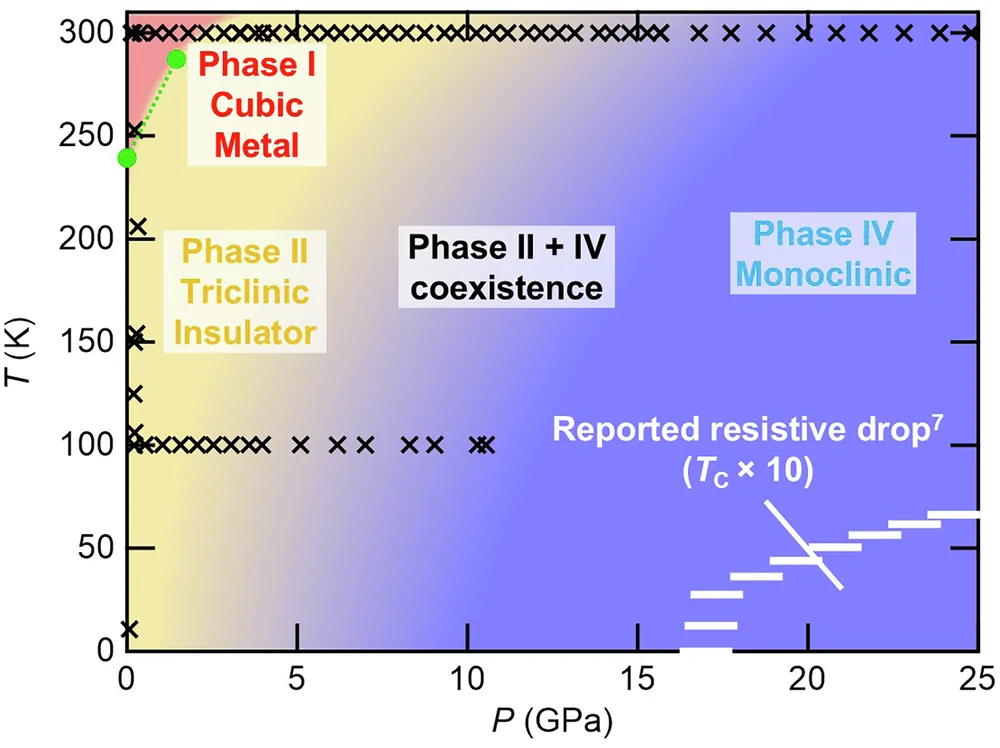
Pressure–temperature phase diagram of CuIr_2_S_4_. Phases I, II, and IV were identified from characteristic changes in the powder X-ray diffraction patterns. The intermediate-pressure region corresponds to two-phase coexistence between Phases II and IV. The structure of Phase II was evaluated by constrained Rietveld refinement of the diffraction dataset collected at 300 K and 7.6 GPa. The Cu and Ir atomic coordinates were refined, whereas the S coordinates and atomic displacement parameters were fixed. Crosses (×) indicate the *P*–*T* conditions where X-ray diffraction data were collected. Green circles mark the Phase-I-to-Phase-II transition temperatures determined from our electrical resistivity measurements. The “Metal” and “Insulator” labels for Phases I and II, respectively, are assigned based on our resistivity data. The insulating behavior was directly established up to 12.6 GPa, within Phase II and the Phase-II + IV coexistence region; the transport character of single-phase IV was not determined here. The low-temperature resistivity drop reported at higher pressures is indicated based on ref. ^7^ for reference; it was not examined in the present transport measurements.

**Fig. 2:**
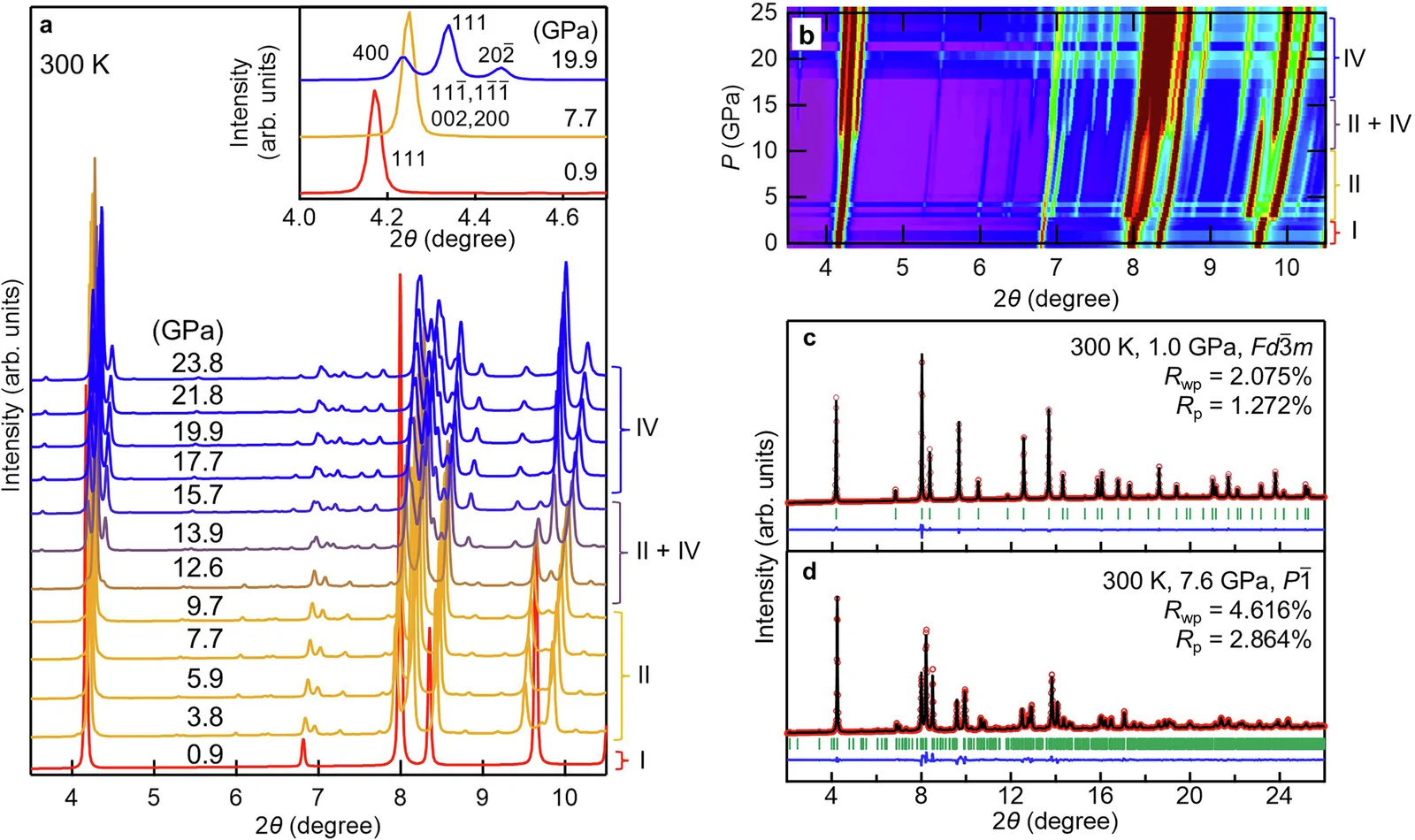
High-pressure powder X-ray diffraction of CuIr_2_S_4_ at room temperature. **a** Pressure evolution of diffraction patterns measured at 300 K. The inset shows an enlarged view of the pressure dependence of the pseudocubic (111) reflection. **b** Color map summarizing the pressure evolution of the diffraction intensity. **c** Rietveld refinement of the cubic phase (Phase I). **d** Constrained Rietveld refinement of Phase II at 300 K and 7.6 GPa. The ambient-pressure low-temperature single-crystal structure reported in ref. ^15^ was used as the initial model. The Cu and Ir atomic coordinates were refined, whereas the S coordinates and atomic displacement parameters were fixed. Red circles, observed intensities; black line, calculated profile; blue line, difference curve; green ticks, Bragg peak positions.

Figure 1 summarizes the pressure–temperature phase diagram of CuIr_2_S_4_ constructed from our diffraction and transport data, highlighting the successive structural changes and the Phase II + IV coexistence regime. We first present this global framework (Fig. 1) and then show representative diffraction data that delineate the corresponding structural evolution (Fig. 2). Figure 2a displays the room-temperature synchrotron powder X-ray diffraction patterns as a function of pressure. With increasing pressure, distinct changes in the diffraction profiles are observed, and the data reveal three distinct phases (I, II, and IV), together with an intermediate two-phase coexistence region of Phases II and IV. The intermediate-pressure region previously referred to as Phase III in ref. ^7^ is therefore identified here as a Phase-II + IV coexistence region rather than a distinct single phase. In our data, the Phase II:Phase IV fraction is approximately 0.303(3):0.697(2) at 300 K and 14.2 GPa, and a similar ratio is observed around 100 K and 10.1 GPa (Supplementary Note 3). These observations are consistent with a dome-like stability region of Phase IV, as illustrated conceptually in Fig. 1.

Figure 2b summarizes the pressure evolution of the diffraction intensity observed in the first experimental run. This color map provides an overview of the structural evolution across the measured pressure range and clearly visualizes the appearance of the Phase II + IV coexistence regime. The first run was used primarily to trace the overall phase evolution and the associated changes in the diffraction profiles. Final structure determination of Phase IV was carried out separately using single-phase datasets collected in a second experimental run at 19.8 and 25.7 GPa under optimized acquisition conditions, providing sufficient signal-to-noise ratios for full-atom refinement.

Figure 2c shows the powder diffraction pattern of Phase I. No peaks originating from impurities were observed, and the diffraction pattern was successfully fitted by assuming the cubic structure with the space group *Fd*$\bar{3}$*m*. Figure 2d shows the constrained Rietveld refinement of Phase II at 300 K and 7.6 GPa. The ambient-pressure low-temperature single-crystal structure reported in ref. ^15^ was used as the initial model. The Cu and Ir atomic coordinates were refined, whereas the S coordinates and atomic displacement parameters were fixed to limit parameter correlations. The calculated profile reproduces both the dominant fundamental reflections and the weaker superlattice reflections, providing an experimentally constrained description of the Cu/Ir sublattice in Phase II under pressure. These results indicate that the structural phase transition temperature increases under pressure and reaches above room temperature. The stabilization of the ambient-pressure low-temperature ordered phase by pressure is consistent with the positive pressure coefficient of the transition temperature expected from the Clausius–Clapeyron relation, given the reported entropy decrease and volume contraction at the transition^20–22^.

**Fig. 3:**
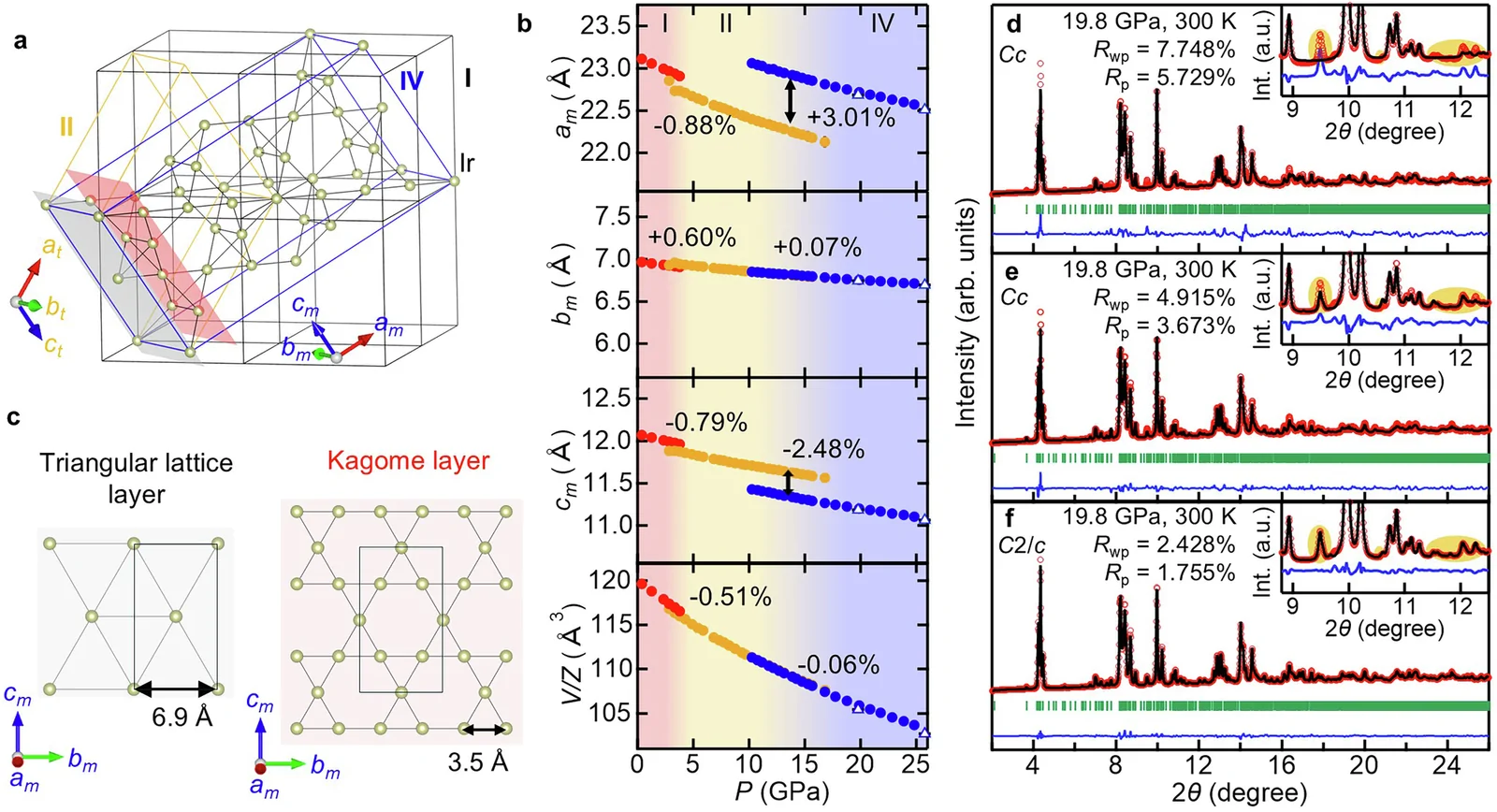
Structural determination of Phase IV. **a** Relationship among the unit cells of Phases I, II, and IV. In the Phase-IV setting, the Ir kagome and triangular layers alternate along the monoclinic *a*_*m*_ axis. **b** Pressure dependence of the lattice parameters, with the unit cells of Phases I and II transformed into the Phase-IV unit-cell setting. **c** Ir triangular- and kagome-layer sublattices in Phase IV, together with representative Ir–Ir distances. **d** Calculated diffraction profile for a trial structural model constructed using the Phase-IV lattice parameters while retaining the internal coordinates of Phase I (space group *Cc*; no atomic-coordinate refinement). **e** Intermediate refinement in space group *Cc*, in which selected Ir coordinates were refined to construct a diffraction-informed starting model and identify the short-bond topology of Phase IV. **f** Final Rietveld refinement of the single-phase Phase-IV dataset collected at 300 K and 19.8 GPa. Starting from the DFT-relaxed structural model, the atomic coordinates of all constituent elements were refined in space group *C*2/*c*. Panels **d**–**f** show the full measured 2θ range, and the insets show enlarged views of the ${8.8}^{\circ }$–${12.5}^{\circ }$ region, where the progressive improvement of the fit is most clearly visible. A quantitative atom-by-atom comparison between the DFT-relaxed and experimentally refined Phase-IV structures is provided in the Supporting Information.

Figure 3a shows the unit cell obtained from Le Bail analysis of the diffraction pattern of Phase IV at 19.8 GPa. For comparison, the unit cells of Phases I and II are also shown. A key point is that the unit-cell volume of Phase IV is approximately twice that of Phase II. Consequently, even when the metric symmetry appears higher than that of Phase II, additional reflections can arise as superlattice peaks associated with the enlarged cell. In our data, the pseudocubic (111) reflection exhibits a clear multi-peak splitting (inset of Fig. 2a), indicating a deviation from orthorhombic or higher metric symmetry and supporting a monoclinic distortion. On the basis of indexing and systematic absences, the space group of Phase IV is constrained to *C*2*/c* or its subgroup *Cc*.

Figure 3b compares the pressure evolution of the lattice parameters across the measured structural regimes, converted to the Phase-IV unit-cell setting and provides important clues to the bonding geometry of Phase IV. In the transition from Phase I to II, *a*_*m*_ and *c*_*m*_ decrease rapidly, resulting in a maximum volume contraction of 0.51%. This contraction is consistent with Ir–Ir dimer formation and the associated bond-disproportionation pattern described within an idealized nominal-valence picture. In the transition from Phase II to IV, *a*_*m*_ increases sharply whereas *c*_*m*_ decreases, and the net volume change is very small. The persistence of the compressed volume suggests that dimers remain present in Phase IV. At the same time, the increase in *a*_*m*_ and decrease in *c*_*m*_ indicate that the short Ir–Ir bonds are formed within the *b*_*m*_ – *c*_*m*_ plane.

As shown in Fig. 3a, the unit cell of Phase IV consists of alternating Ir kagome and triangular layers stacked along the *a*_*m*_ direction. Figure 3c shows that Ir–Ir distances within the triangular layers are significantly longer than those in the kagome layers, suggesting that the short Ir–Ir bonds are primarily formed within the kagome layers. To construct an experimentally guided starting model for Phase IV, we first carried out an intermediate refinement in the subgroup *Cc*. In this step, only selected Ir coordinates, namely the *y* and *z* components of the Ir sites in the kagome-layer sublattice, were refined. This procedure yielded a stable improvement of the fit, as shown by the comparison between Fig. 3d and 3e, and identified the short-bond topology required by the diffraction data.

Starting from this diffraction-informed model, we then performed DFT-based structural optimization while keeping the Bravais lattice type fixed. The relaxed structure converged to a model consistent with *C*2/*c* and was subsequently used as the initial structure for final Rietveld refinements of the single-phase Phase IV datasets collected at 19.8 and 25.7 GPa. Importantly, for both datasets, the atomic coordinates of all constituent elements could be refined stably without divergence. The two refinements consistently reproduce the Phase-IV symmetry and the characteristic Ir–Ir short-bond topology. Figure 3f shows the representative full-atom refinement for the 19.8 GPa dataset over the full measured 2*θ* range. The refined profile reproduces the experimental diffraction pattern well, including both the dominant fundamental reflections and the weaker diagnostic reflections relevant to the Phase IV structure determination. The insets in Fig. 3d–f highlight the 8.8°–12.5° region, where the improvement of the fit can be followed most clearly. The independent refinements at 19.8 and 25.7 GPa consistently establish the Phase-IV symmetry and Ir–Ir short-bond topology, demonstrating that the proposed structure is supported by full-atom refinement rather than solely by a constrained DFT-assisted model. The corresponding refinement profile and refined structural parameters for the 25.7 GPa dataset are provided in the Supporting Information as an independent consistency check.

### Discussion of the structural differences between Phases II and IV

**Fig. 4:**
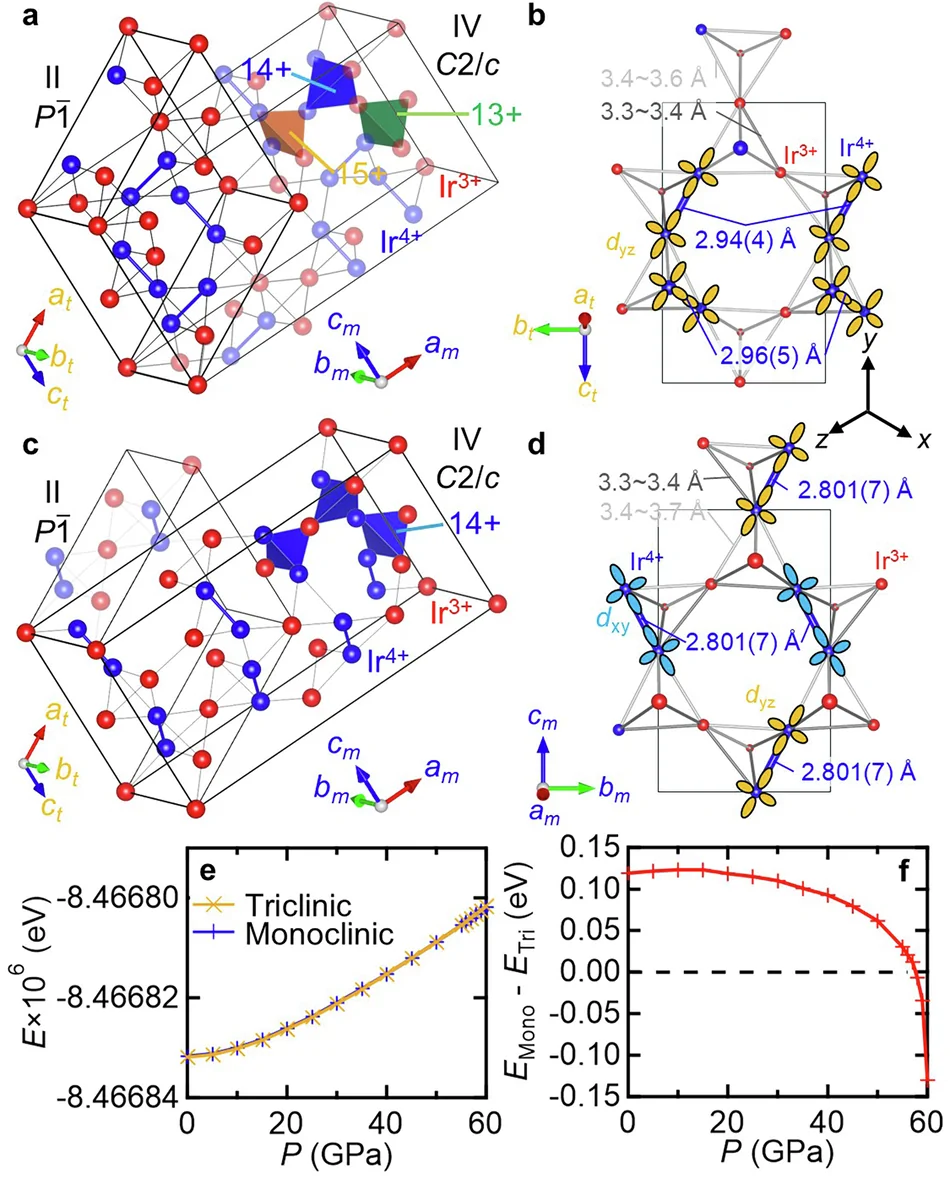
Comparison between Phase II and Phase IV: bond/charge pattern, orbital character, and structural energetics. **a** Bond/charge-disproportionation (dimer) pattern of Phase II. Transparent atoms and the unit cell indicate the corresponding Phase IV pattern (same atomic positions shown for comparison). **b** Schematic relationship between dimers and their dominant orbital characters in Phase II. **c** Bond/charge-disproportionation (dimer) pattern of Phase IV. Transparent atoms and the unit cell indicate the corresponding Phase II pattern. **d** Schematic relationship between dimers and their dominant orbital characters in Phase IV. **b** and **d** depict a three-layer slab consisting of a central kagome layer and its two adjacent triangular layers. As drawn, the larger and smaller Ir spheres correspond to the triangular layers above and below the kagome layer, respectively. The Ir–Ir distances shown in **b** and **d** were obtained from the constrained Rietveld refinement of Phase II at 7.6 GPa and the full-atom Rietveld refinement of Phase IV at 19.8 GPa, respectively. The tetrahedral values shown in **a**–**d** represent sums of nominal Ir³⁺/Ir⁴⁺ assignments used solely to describe the short-bond topology within an idealized ionic picture; they are not bond-valence sums derived from the refined Ir–S coordination environments and do not imply complete integer charge separation. **e** Structural energetics from first-principles calculations. **f** Energy difference Δ*E* = *E*_Mono_ - *E*_Tri_ (per formula unit) as a function of pressure. The dashed line indicates Δ*E* = 0.

The structures of Phases II and IV are compared in Fig. 4a–d. In both phases, the Ir sublattice exhibits pronounced bond disproportionation, with particularly short Ir–Ir bonds defining the dimerized sites. However, the spatial arrangement of these short bonds differs substantially between the two phases. The Phase-II pattern is consistent with the orbitally induced Peierls picture proposed for the ambient-pressure low-temperature phase, in which orbital-selective quasi-one-dimensional chains select a characteristic dimer arrangement. By contrast, Phase IV contains short Ir–Ir bonds along two distinct chain directions and exhibits a different bond-order topology that is not captured by the same simple one-dimensional Peierls description.

To compare the two short-bond patterns, we assign nominal Ir^3+^ and Ir^4+^ labels solely as a bookkeeping scheme within an idealized ionic picture. These labels do not imply complete integer charge separation, and the tetrahedral values shown in Fig. 4a–d are not bond-valence sums calculated from the refined Ir–S coordination environments. Within this nominal-valence representation, the Ir tetrahedra in Phase II have nonuniform sums ranging from 13+ to 15 + , whereas all tetrahedra in Phase IV have a nominal sum of 14 + . The Phase-IV pattern is therefore consistent with an Anderson-type local tetrahedron rule^17–19^. Although this rule is conventionally associated with minimizing intersite Coulomb energy, the present structural analysis does not identify the microscopic interaction responsible for stabilizing Phase IV.

Figure 4e and 4f compare the calculated energies of the Phase-II- and Phase-IV-type structural models. Although the calculated crossover pressure should not be interpreted as a quantitative prediction of the experimental transition pressure, the calculations reproduce a pressure-induced reversal of their relative stability. This result supports the structural interpretation that compression favors the Phase-IV-type short-bond topology over the orbitally selected Phase-II pattern.

### Measurement of electrical resistivity under high pressure

**Fig. 5:**
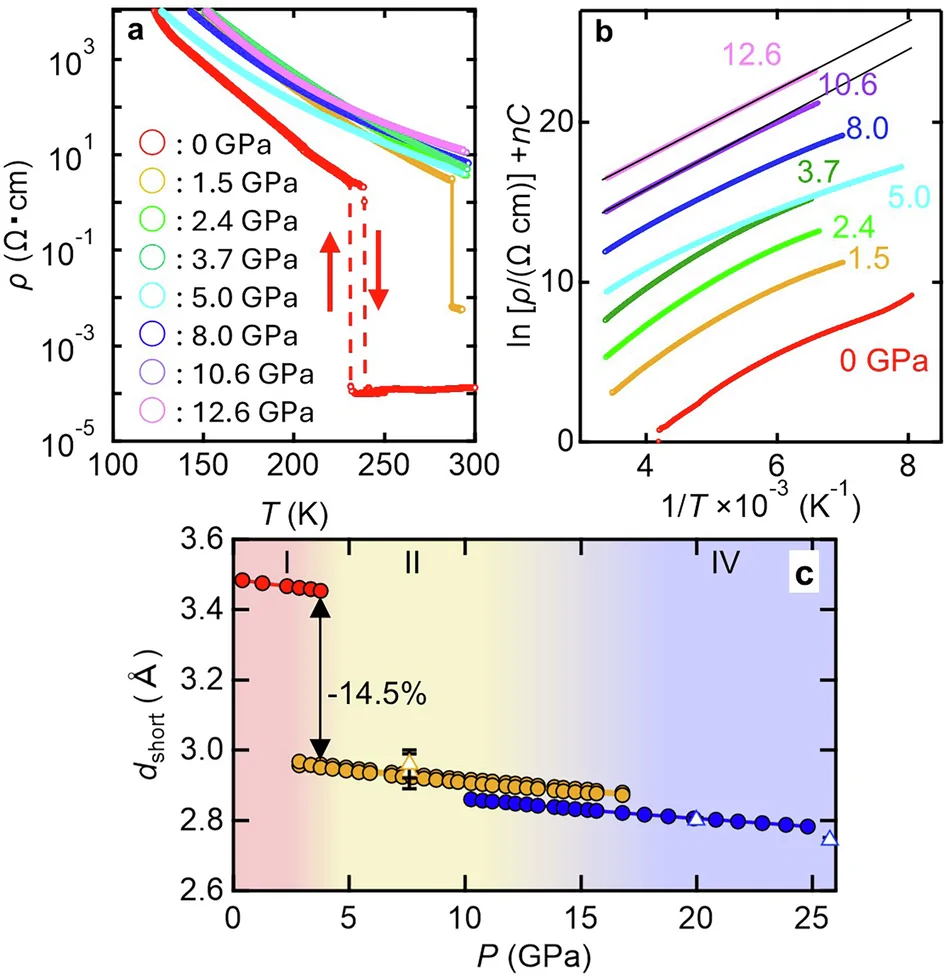
Electrical resistivity and Ir–Ir bond disproportionation under pressure. **a** Temperature dependence of the electrical resistivity at various pressures. **b** Arrhenius representation of the electrical resistivity, plotted as ln[*ρ* /(Ω cm)] + *nC* versus 1/*T*, where *C* = 2 and n is an integer that increases successively with pressure. The constant vertical offsets are introduced only for clarity and do not affect the slopes. Solid lines represent linear fits only to the data at 10.6 and 12.6 GPa, for which sufficiently extended linear regions are observed. No activation energies were extracted from the lower-pressure curves because their linear regions were insufficient for reliable quantitative fitting. **c** Pressure dependence of the shortest nearest-neighbor Ir–Ir distance, *d*_short_, used as a structural measure of Ir–Ir bond disproportionation. Red circles show the experimental values for cubic Phase I. Filled orange symbols are fixed-coordinate Phase-II reference values calculated from the fractional coordinates reported by Ohashi et al. and the experimentally determined lattice parameters at each pressure, whereas the open orange symbol at 7.6 GPa was obtained from the constrained Rietveld refinement. Filled blue symbols show the DFT-relaxed Phase-IV values, and open blue symbols show the experimental values obtained from the full-atom Rietveld refinements at 19.8 and 25.7 GPa. Error bars represent the estimated standard uncertainties.

Figure 5a summarizes the electrical resistivity measured under pressure up to 12.6 GPa. High-pressure resistivity measurements on sintered polycrystalline samples have been reported previously^6^, whereas the present measurements were performed on single-crystal samples. At ambient pressure, a pronounced resistivity increase is observed at the metal–insulator transition. With increasing pressure, the room-temperature resistivity increases, and at 2.4 GPa, corresponding to Phase II, an insulating temperature dependence extends to room temperature. These transport results form the basis for the metallic Phase-I and insulating Phase-II assignments in Fig. 1, while the transport character of single-phase Phase IV was not directly determined here.

Figure 5b presents the Arrhenius representation of the electrical resistivity. The datasets obtained at 10.6 and 12.6 GPa exhibit sufficiently extended linear regions in the high-temperature range to permit quantitative Arrhenius fits. The corresponding apparent activation energies obtained from the linear fits are 0.189 eV at 10.6 GPa and 0.186 eV at 12.6 GPa. By contrast, the lower-pressure curves show only limited or curved regions and were therefore not used to extract activation energies. Accordingly, the present analysis establishes a clearly activated temperature dependence at 10.6 and 12.6 GPa, but does not provide a reliable pressure dependence of the activation energy over the lower-pressure region.

Figure 5c summarizes the pressure evolution of the shortest Ir–Ir nearest-neighbor distance, *d*_short_, as a structural measure of Ir–Ir bond disproportionation. For Phase II, the experimental *d*_short_ value at 7.6 GPa was evaluated from the Ir coordinates obtained by the constrained Rietveld refinement. The orange symbols were calculated by retaining the fractional atomic coordinates of the ambient-pressure low-temperature structure reported by Ohashi et al. and substituting the experimentally determined lattice parameters at each pressure. These values provide a reference for the change in *d*_short_ expected solely from the evolution of the lattice parameters, without internal-coordinate relaxation. For Phase IV, the experimental values were obtained from the full-atom Rietveld refinements of the single-phase datasets collected at 19.8 and 25.7 GPa, whereas the blue symbols represent the corresponding values derived from the DFT-relaxed initial models. The experimentally refined values confirm the presence of short Ir–Ir bonds in both ordered phases, while establishing that Phases II and IV exhibit distinct short-bond topologies.

## Conclusions

In conclusion, we have clarified the crystallographic evolution of CuIr_2_S_4_ up to approximately 25 GPa. For Phase II, constrained Rietveld refinement at 300 K and 7.6 GPa provides an experimentally constrained description of the Cu/Ir sublattice and the Ir–Ir short-bond pattern under pressure. The Cu and Ir coordinates were refined, whereas the S coordinates and atomic displacement parameters were fixed to limit parameter correlations. We identify a two-phase coexistence regime between Phases II and IV and establish the structure of Phase IV from single-phase diffraction datasets collected at 19.8 and 25.7 GPa. Starting from a DFT-relaxed initial model, the atomic coordinates of all constituent elements could be refined stably in space group *C*2/*c* for both datasets, yielding the same characteristic Ir–Ir short-bond topology. The resulting Phase IV structure exhibits a charge/bond ordering pattern distinct from the orbitally induced Peierls-type order in Phase II and is instead consistent with an Anderson-type local tetrahedron rule on the pyrochlore lattice. Together with the total-energy calculations, these results place the pressure-driven order selection in CuIr_2_S_4_ on a firmer crystallographic basis. More broadly, the present work provides a structural foundation for future studies that correlate bond disproportionation, charge/bond rearrangement, and emergent electronic phases across the wider high-pressure phase diagram of frustrated spinel systems.

## Methods

### Sample synthesis

The CuIr_2_S_4_ powder sample was synthesized by a solid-state reaction method. Cu, Ir and S were mixed in stoichiometric ratios, vacuum-sealed in quartz tubes, and sintered at 850 °C for 7 days. The single-crystal samples for high-pressure electrical resistivity measurements were synthesized using the Bi flux method^23^.

### High-pressure powder X-ray diffraction

High-pressure powder X-ray diffraction (XRD) measurements were performed at BL10XU of SPring-8 using monochromatic X-rays with *E* = 30 keV^24^. An imaging plate was used as the detector (R-AXIS IV++), and camera lengths of 300 and 400 mm were used in different experimental runs. The powder sample was loaded into a diamond anvil cell (DAC) together with He as a pressure-transmitting medium. The culet diameter of the diamond anvil was 0.5 mm, and a rhenium gasket was used. Pressure was determined by ruby fluorescence^25,26^.

### Data-analysis workflow: Le Bail and Rietveld analyses

Le Bail fitting was used to determine the lattice parameters and to identify symmetry changes and phase coexistence from characteristic changes in the diffraction patterns across the measured pressure range.

For Phase II, constrained Rietveld refinement was performed using the diffraction dataset collected at 300 K and 7.6 GPa. The ambient-pressure low-temperature single-crystal structure reported in ref. ^15^ was used as the initial model. The Cu and Ir atomic coordinates were refined, whereas the S coordinates and atomic displacement parameters were fixed to the values adopted from the ambient-pressure low-temperature structural model to limit parameter correlations associated with the triclinic *P*$\bar{1}$ structure containing 28 crystallographically independent sites. To estimate the pressure evolution of *d*_short_ within Phase II in the absence of internal-coordinate relaxation, the fractional atomic coordinates of the ambient-pressure low-temperature structure reported in ref. ^15^ were retained, while the experimentally determined lattice parameters at each pressure were substituted.

For Phase IV, the first experimental run was used primarily to establish the overall phase evolution, phase coexistence, and lattice-metric changes across the measured pressure range. On the basis of these results, an experimentally guided starting model was constructed through Le Bail analysis, intermediate refinement of selected Ir coordinates, and subsequent DFT-based structural optimization. Final structure determination of Phase IV was then carried out using the single-phase diffraction datasets collected at 19.8 and 25.7 GPa. For the 19.8 GPa refinement, the fractional atomic coordinates of the DFT-relaxed Phase-IV model were expressed using the experimentally determined lattice parameters at 19.8 GPa and used as the initial structure. For the 25.7 GPa refinement, the fractional atomic coordinates of the same DFT-relaxed model were expressed using the experimentally determined lattice parameters at 25.7 GPa and used as the initial structure. In both refinements, the atomic coordinates of all constituent elements were refined in space group *C*2/*c*. Thus, the first run served to define the phase evolution and to construct a physically motivated starting model, whereas the second run provided the high-quality single-phase datasets used for the final full-atom structure determination.

Le Bail fitting and Rietveld refinement were carried out using RIETAN-FP^27^. Crystal structures and polyhedral drawings were generated using VESTA^28^.

### High-pressure electrical resistivity measurements

Electrical resistivity measurements were carried out using an opposed-anvil cell^29^. The culet size of the anvil was 3 mm, and a NiCrAl gasket was used. Glycerin was used as the pressure-transmitting medium. The pressure was determined from the superconducting transition temperature of lead.

### Calculations based on crystal structure

The structure optimization calculation was performed using density-functional theory with the Perdew-Burke-Ernzerhof generalized-gradient approximation for solids (PBEsol)^30^ and Kresse-Joubert projector-augmented-wave pseudopotentials^31,32^, implemented in the Quantum ESPRESSO package^33,34^. Monkhorst–Pack *k*-point meshes of 8×8×8 and 6×6×6 were used for the monoclinic and triclinic structures, respectively, and the plane-wave cutoff energy was set to 120 Ry. Spin–orbit coupling was not included during the structural optimization but was included in the total-energy calculations.

### Supporting information

Constrained Rietveld refinement and refined structural parameters for Phase II at 300 K and 7.6 GPa; comparison between the refined Phase-II short Ir–Ir distances and fixed-coordinate reference values; the DFT-relaxed Phase-IV structural model and full-atom refinement results at 19.8 and 25.7 GPa; atom-by-atom positional differences between the DFT-relaxed and experimentally refined Phase-IV structures; and additional two-phase fitting results supporting the phase assignments and coexistence regimes discussed in the main text.

## Supplementary information

Transparent Peer Review file

Supplemental Material

## Data Availability

The experimentally refined Phase-IV structure at 19.8 GPa has been deposited with the Cambridge Crystallographic Data Centre under deposition number CCDC 2551065. These data can be obtained free of charge from the Cambridge Crystallographic Data Centre via https://www.ccdc.cam.ac.uk/structures/. The DFT-relaxed initial model and the structural parameters obtained from the 25.7 GPa refinement are provided in the Supporting Information. The remaining data supporting the findings of this study are available from the corresponding author upon reasonable request.
