# Supplementary material for: Crystallographic origin of pressure-induced bond order in the frustrated spinel CuIr2S4
Source: Commun Chem. 2026 Oct 26;9:316. doi: 10.1038/s42004-026-02240-y (PMC13710760; doi:10.1038/s42004-026-02240-y)
Supplement: Supplementary file 1 [file 42004_2026_2240_MOESM1_ESM.pdf]

nature portfolio

Peer Review File

Crystallographic origin of pressure-induced bond order in the frustrated spinel CuIr2S4

Corresponding Author: Professor Naoyuki Katayama

This file contains all reviewer reports in order by version, followed by all author rebuttals in order by version.

Version 0:

Reviewer comments:

Reviewer #1

(Remarks to the Author)
Having reviewed the previous referee report, the rebuttal letter and read the new version, I am satisfied that Referee I's criticism has been fully addressed. This concerned mainly the refinement of Phase 2, which is not the main focus of the present manuscript. However, Referee I was entirely right in requesting that the refinement of Phase 2 should be at least of the same quality as that of Phase 4. The structural differences between these two phases are subtle, hence it is importance to be as confident as possible within the limits imposed by the powder method. Complex ordered phases in spinels are notoriously difficult to pin down and are often periodically revised, but the first refinement of Phase IV of CuIr2S4 is definitely a milestone. There are aspects of this paper I am not entirely satisfied with — in particular, the discussion of the physics. Following the original structural solution, the structure was discussed theoretically as a 'valence bond crystal' (Di Matteo et al. Phys. Rev. B 72, 024431). Such discussion might still be applicable tot he revised structure in Ref 15, but attributing the origin of Phase IV to "the enhanced role of intersite Coulomb interaction" seems somewhat speculative, especially since one would naively expect the opposite. Nevertheless, since this paper is submitted to a chemistry journal (rather than a physics one) I think the lack of a toy-model physical explanation is not critical. I would, however, request that the Authors remove the label "Phase 3" and replace it with "Phase 2-Phase 4 coexistence" (or perhaps even better, "Phase 2-Phase 3 coexistence" since the Phase 3 label is now available. In the ordinary thermodynamic usage, a "phase" is a homogeneous state characterised by its own thermodynamic properties/order parameters. A coexistence region is instead a region where two distinct "phases" coexist. Otherwise, and pending the reception of further reviews, I am happy to recommend in favour of publication in Communications Chemistry.

Open Access This Peer Review File is licensed under a Creative Commons Attribution 4.0 International License, which permits use, sharing, adaptation, distribution and reproduction in any medium or format, as long as you give appropriate credit to the original author(s) and the source, provide a link to the Creative Commons license, and indicate if changes were made.
In cases where reviewers are anonymous, credit should be given to 'Anonymous Referee' and the source.
The images or other third party material in this Peer Review File are included in the article's Creative Commons license, unless indicated otherwise in a credit line to the material. If material is not included in the article's Creative Commons license and your intended use is not permitted by statutory regulation or exceeds the permitted use, you will need to obtain permission directly from the copyright holder.
To view a copy of this license, visit https://creativecommons.org/licenses/by/4.0/

Reviewer #1

In the revised version of the manuscript and in the rebuttal, the authors have responded to some of the points raised in my previous report, however several important points remain unclear or not satisfactory.

(1) The authors do not say either in the paper or in the rebuttal why they used the Le Bail method to fit the diffraction profile of phase II, instead of a full Rietveld refinement, as they did for phase IV.

We thank the reviewer for pointing out that, in the previous version, we did not clearly explain why Phase II was treated by Le Bail/profile-based fitting rather than by Rietveld refinement, whereas Phase IV was analyzed by Rietveld refinement.

The reason is that the Phase-II dataset available at the time of the previous submission did not have sufficient intensity reliability for stable atomic-coordinate refinement. In particular, the strongest low-angle fundamental reflection around 4° dominated the refinement behavior: because its observed intensity could not be reproduced reliably, the refinement was strongly pulled by this reflection and tended to diverge.

After re-examining the data-acquisition conditions, we found that this was not a fundamental failure of the assumed structural model, but a technical problem related to the detector readout. In the imaging-plate system used for this experiment, low- and high-intensity signals are read through separate PMT channels (PMT-L and PMT-H) and then merged into a single output. Under normal conditions, reflections recorded partly through the high-intensity channel should still yield correct merged intensities. The actual problem in the previous Phase-II experiment was that, in the intensity range relevant to the strongest low-angle reflections, the linearity between the PMT-L and PMT-H channels was not properly maintained. As a result, the merged intensities of those reflections became anomalously large, which compromised exactly the reflections most critical for stable structural refinement.

Because users only receive the merged detector output after the PMT-L and

PMT-H channels have been combined, this problem could not be diagnosed directly from the merged intensity output available to users. For this reason, in the previous version we did not regard a Rietveld refinement of Phase II as sufficiently reliable, and instead used profile-based fitting with the previously reported low-temperature single-crystal structure as a structural reference. We agree with the reviewer that this point should have been explained explicitly in our previous rebuttal. Because the revised manuscript no longer uses the earlier Phase-II dataset or the associated profile-based treatment as the basis for the structural analysis, we describe the detector-related issue here in the response letter rather than in the manuscript.

To address this issue, we carried out an additional high-pressure diffraction experiment under revised acquisition conditions. Specifically, we intentionally used a slightly shorter exposure so that all reflections remained within the PMT-L readout range and the measurement no longer depended on the affected PMT-L/PMT-H cross-calibration. This change did not materially degrade the counting statistics of the dataset, but it substantially improved the intensity reliability of the strongest low-angle reflections.

Using this improved dataset, we were able to perform a constrained Rietveld refinement for Phase II. In the revised refinement, the Cu and Ir atomic coordinates were refined, whereas the S coordinates were fixed to the values reported for the low-temperature single-crystal structure. We emphasize that the remaining constraint on the S coordinates reflects the intrinsic limitation of the present DAC powder diffraction data for a triclinic $P\bar{1}$ structure with a large unit cell and many independent sites, rather than the detector-related problem discussed above. Thus, the revised analysis no longer relies only on Le Bail fitting for Phase II.

(2) I understand that the authors focus their attention on the high-pressure phase IV, but their purpose is to establish a relationship between the different crystal structures of phases II and IV and subtle phenomena, such as dimerization, charge and orbital orderings. So, they cannot dismiss the need for a precise knowledge of the pressure-induced changes in the pristine phase II. In view of this, a Le Bail fit is not appropriate, for the crystal structure of phase II is very complex: it exhibits the lowest possible triclinic symmetry, so all atoms are located in general positions, and contains as many as 28 nonequivalent sites. Therefore, for a comparison with phase IV, it is not legitimate to assume that the atomic positions in phase II are unchanged under high pressure. On the contrary, I would expect that pressure affects important structural parameters governing the above phenomena, such as the Ir-Ir bond distances and bond valence sums.

We thank the reviewer for this important comment. We agree that the Phase-II treatment based only on Le Bail/profile-based fitting in the previous version was not sufficient for a detailed comparison with Phase IV at the level of structural quantities such as dimerization-related Ir–Ir distances. We also agree that it is not appropriate to assume that all atomic positions in Phase II remain unchanged under pressure.

To address this concern, we have reanalyzed Phase II using the newly acquired diffraction dataset described in our response to Comment (1). In the revised analysis, we no longer use the fixed-coordinate treatment adopted in the previous version. Instead, we perform a constrained Rietveld refinement in which the Cu and Ir atomic coordinates are refined against the experimental high-pressure powder-diffraction data. Thus, the revised Phase-II model is experimentally constrained under pressure and no longer assumes that the Cu and Ir positions remain unchanged from those of the ambient-pressure low-temperature structure.

At the same time, a fully unconstrained refinement of all atomic coordinates is intrinsically difficult for the present Phase-II dataset. Phase II has triclinic $P\bar{1}$ symmetry, a large unit cell, and 28 crystallographically independent sites, all

located at general positions. In addition, the present data were obtained from a small sample in a diamond-anvil cell, and S contributes much less strongly to the X-ray diffraction intensity than Ir and Cu. Releasing all S coordinates together with the Cu and Ir coordinates therefore results in an unstable and highly correlated refinement. We have consequently fixed the S coordinates to those determined for the ambient-pressure low-temperature single-crystal structure, while refining the Cu and Ir coordinates. The atomic displacement parameters were fixed to the values adopted from the ambient-pressure structural model.

This treatment is consistent with the known experimental difficulty of determining the complete Phase-II structure from powder diffraction alone. In the study by Ohashi et al. [Ref. 15], the definitive atomic coordinates of the ambient-pressure low-temperature Phase-II structure were determined from single-crystal synchrotron X-ray diffraction. The subsequent powder X-ray and neutron Rietveld analyses used the single-crystal-derived structure as the initial model and showed almost no change in the structural parameters. These powder analyses therefore served primarily as independent consistency checks rather than as an unconstrained structure solution from powder data alone. Ohashi et al. also explicitly noted that precise Rietveld refinement of this low-symmetry structure from powder-diffraction data is extremely difficult.

We therefore do not claim that the present high-pressure Phase-II structure has been determined by a fully unconstrained all-atom refinement. Rather, the revised analysis provides an experimentally constrained basis for evaluating the pressure-induced changes of the Cu/Ir sublattice and for comparing its bond pattern with that of Phase IV. In particular, the Ir–Ir distances in Phase II are now calculated from experimentally refined Ir coordinates rather than from an unchanged ambient-pressure reference structure.

Regarding bond-valence sums, we agree that they depend sensitively on the complete set of ligand coordinates. Because the S coordinates remain fixed in the present refinement, we no longer use bond-valence sums as a primary basis for comparing Phases II and IV. Instead, the revised manuscript focuses on the quantities directly constrained by the present data, namely the Ir

sublattice, the topology of the short Ir–Ir bonds, and the pressure evolution of the dimerization pattern.

Thus, although the revised Phase-II refinement necessarily retains constraints reflecting the intrinsic limitations of DAC powder diffraction, it no longer relies on Le Bail fitting alone and no longer assumes unchanged transition-metal coordinates under pressure. We believe that this provides a substantially firmer and appropriately qualified crystallographic basis for comparison with Phase IV.

(3) As a matter of fact, the Le Bail fit for phase II yields significant intensity difference in the most intense peaks, as seen in Supplementary Figure 1a, which raises a concern as to reliability of the structural model used for phase II under pressure.

We thank the reviewer for this important comment. We agree that the significant intensity mismatch at the strongest reflections in Supplementary Figure 1a of the previous version raised a legitimate concern about the reliability of the Phase-II structural model under pressure. In general, an intensity mismatch in a powder-diffraction profile can reflect an inadequacy of the assumed internal structure, even when the peak positions and lattice parameters are reproduced. Indeed, in the ambient-pressure study by Ohashi et al. [Ref. 15], the previously reported Phase-II model reproduced the peak positions but not the intensities, whereas the structure determined from single-crystal diffraction provided a substantially improved fit to the powder-diffraction pattern. We therefore agree that the discrepancy in our previous dataset could not simply be disregarded.

As explained in our response to Comment (1), we subsequently re-examined the acquisition conditions and identified a detector-readout problem affecting the strongest low-angle reflections. In the previous Phase-II measurement, the linearity between the PMT-L and PMT-H readout channels was not properly maintained in the relevant intensity range, resulting in anomalously large merged intensities for these reflections. Nevertheless, the previous data alone did not allow us to distinguish unambiguously between this technical effect and a possible inadequacy of the structural model.

We therefore carried out an additional high-pressure diffraction experiment under revised acquisition conditions. A slightly shorter exposure was used so that all reflections remained within the PMT-L readout range, thereby avoiding the problematic PMT-L/PMT-H crossover. This change did not materially degrade the counting statistics but substantially improved the intensity reliability of the strongest low-angle reflections.

Using the newly acquired dataset, we performed a constrained Rietveld refinement of Phase II over the full measured diffraction range. The Cu and Ir atomic coordinates were refined, whereas the S coordinates were fixed to those of the ambient-pressure low-temperature single-crystal structure. Importantly, the strongest fundamental reflections were retained in the refinement, and their intensities, together with the weaker superlattice reflections, are now reproduced substantially better. The revised refinement profile is shown in Fig. 2d of the main text, and the refined lattice and atomic parameters are provided in Supplementary Table 1.

The new measurements show that the dominant intensity mismatch in the previous high-pressure dataset can be accounted for by the detector-readout problem without requiring a different Phase-II ordering topology. Accordingly, the previous Le Bail/profile-based fit shown in Supplementary Figure 1a is no longer used as the structural basis for Phase II and has been replaced by the new full-profile constrained Rietveld analysis. We believe that this revised analysis directly addresses the reviewer's concern regarding the reliability of the Phase-II structural model.

(4) Related to the above point, methodologically it is not acceptable to remove the most intense peaks, where the experimental profile deviates significantly from the model, to refine the Ir positions (see Supplementary Figure 1b). Indeed, I presume that the most intense peaks are precisely those where the Ir contribution is largest.

We thank the reviewer for this important comment. We agree that, in the previous version, it was methodologically unsatisfactory to exclude the strongest fundamental reflections and then use the resulting Ir-only refinement as a

crystallographic basis for discussing Phase II. We also agree that the strongest reflections are likely to contain important information on the Ir sublattice and therefore should not be omitted in the final structural analysis.

The analysis shown in Supplementary Figure 1b of the previous version was introduced only as an intermediate workaround at a stage when the intensity anomaly of the strongest low-angle reflections had not yet been understood. As clarified by our re-examination of the data-acquisition conditions, those reflections were affected by a detector-readout problem that caused anomalously large merged intensities in the previous Phase-II dataset. Under those circumstances, excluding the most severely affected reflections was an attempt to test whether a partially constrained Ir-sublattice model could still be extracted from the remaining data. However, we agree with the reviewer that such a treatment is not appropriate as the basis for the final structural discussion.

For this reason, in the revised manuscript we no longer use the previous Ir-only refinement after excluding intense reflections. Instead, we use a newly acquired diffraction dataset with substantially improved intensity reliability and perform a constrained Rietveld refinement of Phase II using the full measured diffraction profile. In this revised refinement, the strongest fundamental reflections were retained and no reflections were excluded. The Cu and Ir atomic coordinates were refined, whereas the S coordinates and atomic displacement parameters were fixed to the values adopted from the ambient-pressure low-temperature structural model. The revised refinement profile is shown in Fig. 2d of the main text, and the refined lattice and atomic parameters are provided in Supplementary Table 1.

Thus, we agree with the reviewer that the previous exclusion-based Ir-only treatment should not be used as the structural basis for Phase II. The revised manuscript removes that treatment from the structural argument and replaces it with a constrained Rietveld refinement based on a new dataset in which the strongest reflections are properly included. We believe that this revision resolves the methodological concern raised by the reviewer.

(5) Supplementary Tables 3 and 4 contain the atomic parameters of the calculated and refined structures, but the lattice parameters should be given as well.

We thank the reviewer for this comment. We agree that the lattice parameters should be provided together with the atomic parameters.

In the revised Supporting Information, we have added the corresponding lattice parameters to Supplementary Tables 3 and 4. The table for the calculated structure now includes the unit-cell parameters used for that structure, and the table for the refined structure now includes the refined lattice parameters together with the atomic coordinates. This revision makes the crystallographic information self-contained and easier to follow.

(6) In Supplementary Table 3, where the authors report the calculated structure, they give also the thermal parameters (identical to the refined ones!) which makes no sense.

We thank the reviewer for this comment. We agree that the thermal parameters should not have been listed for the calculated structure in Supplementary Table 3. Their inclusion in the previous version was inappropriate, especially because the listed values were identical to those of the refined structure and therefore did not have an independent physical meaning for the calculated model.

In the revised Supporting Information, we have removed the thermal parameters from Supplementary Table 3. The revised table now lists only the crystallographic parameters relevant to the calculated atomic positions, together with the corresponding lattice parameters. We believe that this revision resolves the reviewer's concern.

(7) In Supplementary Table 4, the statistical uncertainty of the thermal parameters is missing and the numerical values contain an excessive number of digits.

We thank the reviewer for this comment. We agree that the numerical values in Supplementary Table 4 contained an excessive number of digits in the previous version, and that the presentation of the thermal parameters was not sufficiently clear.

In the Phase-IV refinement, the atomic displacement parameters were not refined independently for every crystallographic site. Instead, they were constrained by groups: the displacement parameters were constrained to be identical within each element species, except for Ir, for which the sites involved in dimer formation and those not involved in dimer formation were treated as two separate groups. Therefore, listing site-by-site thermal parameters without clearly indicating the corresponding constraint and uncertainty was not an appropriate presentation.

In the revised Supporting Information, we have rounded the numerical values to an appropriate number of significant digits. We have also revised the presentation of the thermal parameters so that it reflects the actual refinement procedure more faithfully. Specifically, the constraint on the displacement parameters is now explicitly stated in the table caption, and the refined group values are reported in a form consistent with the constrained refinement. The corresponding statistical uncertainties are now given for the refined groups, rather than being implied as independent site-by-site uncertainties. Because the atomic displacement parameters were refined as shared parameters within each constrained group, all sites belonging to the same group share the same refined value and estimated standard uncertainty. In the revised table, the same value and uncertainty are therefore repeated for each site in the corresponding group, and the grouping constraints are explicitly stated in the table caption.

(8) To show an agreement between calculated and refined structures, the authors should give the distance between calculated and refined positions or use appropriate programs, such as the PSEUDO and STRAIN programs of the Bilbao Crystallographic Server.

We thank the reviewer for this helpful suggestion. We have adopted the first of the two alternatives proposed by the reviewer and added a direct atom-by-atom comparison between the calculated and refined structures. After expressing both structures in the same crystallographic setting, matching the corresponding symmetry-equivalent sites, and using the same origin convention, we calculated the real-space displacement of each corresponding atomic position. For the representative 19.8 GPa dataset, the individual positional differences, together with the maximum and root-mean-square positional differences, are now listed in Supplementary Table 4. The corresponding site-by-site comparison at 25.7 GPa is provided in Supplementary Table 5 as an independent consistency check.

The somewhat larger positional deviations for Cu and S than for Ir may partly reflect their weaker X-ray scattering contributions and correspondingly larger positional uncertainties in the DAC powder refinement. The Ir sublattice, which is central to the present discussion of dimer formation and bond disproportionation, shows closer agreement between the calculated and refined structures. We have clarified this distinction and avoid using the Cu and S agreement as the primary basis of the structural argument.

We believe that this direct real-space comparison fulfills the reviewer’s request and provides a transparent quantitative assessment of the agreement between the calculated and experimentally refined structures.

(9) In the resistivity curves of Figure 5b, an Arrhenius behavior is seen only in the two curves measured at 12.6 and 10.6 GPa; the remaining curves measured at lower pressures do not show any linear behavior, so it is not meaningful to extract the activation energy for these curves.

We thank the reviewer for this important comment. We agree that the lower-

pressure resistivity curves do not exhibit sufficiently extended linear regions in the Arrhenius representation to justify the extraction of well-defined activation energies. In the previous version, effective activation energies were estimated from limited temperature intervals; however, we recognize that this treatment could give the misleading impression that a simple thermally activated conduction mechanism is established over the entire pressure range.

In the revised manuscript, we have therefore removed the activation energies extracted from the lower-pressure curves and all discussion based on those values. Quantitative Arrhenius fits are now restricted to the data at 10.6 and 12.6 GPa, for which clear linear regions are observed. The fitting ranges are indicated by the solid fitting lines in the revised figure. The corresponding apparent activation energies obtained from these fits are 0.189 eV at 10.6 GPa and 0.186 eV at 12.6 GPa.

We have also revised Fig. 5b so that the vertical axis is labeled as $\ln[\rho/(\Omega\,\mathrm{cm})] + nC$, where $C = 2$ and n is an integer that increases successively with pressure. The individual curves are vertically offset only for clarity, and the offsets do not affect their slopes. Linear fitting lines are now shown only for the 10.6 and 12.6 GPa datasets.

Accordingly, the revised manuscript no longer uses the lower-pressure activation energies to support the structural or electronic interpretation. The discussion is now limited to the observation that the resistivity exhibits a clearly activated temperature dependence at 10.6 and 12.6 GPa.

(10) In Figure 5b, why did the authors give the scale of the vertical axis in arbitrary units if they measured the absolute value of the resistivity in Figure 5a?

We thank the reviewer for pointing this out. The resistivity was measured on an absolute scale, as shown in Fig. 5a. In the previous version of Fig. 5b, the vertical axis was labeled in arbitrary units because the individual Arrhenius curves had been shifted vertically for clarity. We agree that this labeling was misleading because it could give the impression that the absolute resistivity values were not

available.

In the revised figure, we have retained a numerical vertical scale and explicitly labeled the plotted quantity as $\ln[\rho/(\Omega\,\mathrm{cm})] + nC$, where $C = 2$ and n is an integer that increases successively with pressure. The curves are therefore separated by a constant vertical offset of 2 for clarity. This procedure and the value of the offset are now explicitly stated in the figure caption. The revised presentation makes clear that the underlying measurements were performed on an absolute resistivity scale and that only the graphical positions of the curves were shifted.

(11) The text of the manuscript is difficult to read and several parts are not clear, especially the discussion about the structural differences between phases II and IV and the conclusions.

We thank the reviewer for this important comment. We agree that the presentation of the structural differences between Phases II and IV, as well as the conclusions drawn from this comparison, was not sufficiently clear in the previous version.

We have therefore substantially reorganized and rewritten the manuscript. First, the definitions and structural characteristics of Phases II and IV are now introduced explicitly before their comparison. Second, we have separated the experimentally established structural results from their physical interpretation and have clarified which conclusions are directly supported by the diffraction data. Third, the discussion of charge order, orbital order, and dimerization has been revised to avoid overinterpretation, particularly where the ligand coordinates are not independently refined. We have also removed or softened speculative statements concerning superconductivity that were not directly supported by the present structural analysis.

Finally, the Conclusions section has been rewritten to summarize separately (i) the experimentally determined high-pressure structures, (ii) the structural distinction between Phases II and IV, and (iii) the implications and limitations of the present analysis. The manuscript has also been edited throughout to improve its logical flow, terminology, and readability.

We believe that these revisions have substantially improved the clarity of both the structural discussion and the conclusions.

Reviewer #1

Having reviewed the previous referee report, the rebuttal letter and read the new version, I am satisfied that Referee I's criticism has been fully addressed. This concerned mainly the refinement of Phase 2, which is not the main focus of the present manuscript. However, Referee I was entirely right in requesting that the refinement of Phase 2 should be at least of the same quality as that of Phase 4. The structural differences between these two phases are subtle, hence it is important to be as confident as possible within the limits imposed by the powder method. Complex ordered phases in spinels are notoriously difficult to pin down and are often periodically revised, but the first refinement of Phase IV of $CuIr_2S_4$ is definitely a milestone.

We sincerely thank the reviewer for carefully examining the revised manuscript and for recognizing the significance of the Phase-IV refinement. We are pleased that the additional Phase-II measurement and constrained Rietveld refinement have resolved the principal concern raised in the previous review round.

(1) There are aspects of this paper I am not entirely satisfied with—in particular, the discussion of the physics. Following the original structural solution, the structure was discussed theoretically as a ‘valence bond crystal’ (Di Matteo et al. Phys. Rev. B 72, 024431). Such discussion might still be applicable to the revised structure in Ref. 15, but attributing the origin of Phase IV to ‘the enhanced role of intersite Coulomb interaction’ seems somewhat speculative, especially since one would naively expect the opposite. Nevertheless, since this paper is submitted to a chemistry journal (rather than a physics one), I think the lack of a toy-model physical explanation is not critical.

We thank the reviewer for this important comment. We agree that the present structural data and total-energy calculations do not, by themselves, identify the microscopic interaction responsible for selecting Phase IV. We have therefore removed the attribution to an enhanced role of intersite Coulomb interactions from the Abstract, the final paragraph of the Introduction, and the Conclusions. We now restrict our interpretation to the crystallographic observation that the Phase-IV bond pattern is consistent with an Anderson-type local tetrahedron rule.

(2) I would, however, request that the Authors remove the label ‘Phase 3’ and replace it with ‘Phase 2–Phase 4 coexistence’ (or perhaps even better, ‘Phase 2–Phase 3 coexistence’ since the Phase 3 label is now available). In the ordinary thermodynamic usage, a ‘phase’ is a homogeneous state characterised

by its own thermodynamic properties/order parameters. A coexistence region is instead a region where two distinct 'phases' coexist.

We fully agree. We have removed Phase III as the designation of a distinct crystallographic phase and now identify the intermediate-pressure region consistently as the Phase II + IV coexistence region throughout the main text, figures, figure captions, and Supporting Information. We retained the name Phase IV for the homogeneous high-pressure phase to preserve continuity with the nomenclature used in Ref. 7 and to avoid ambiguity when comparing the present results with the previously published phase diagram. The revised manuscript explicitly states: "The intermediate-pressure region previously referred to as Phase III in Ref. 7 is therefore identified here as a Phase-II + IV coexistence region rather than a distinct single phase." Corresponding changes have been made in Figs. 1 and 2 and in Supplementary Fig. 3, as well as in all associated descriptions and captions.

We thank the reviewer again for the constructive comments, which have helped us make both the physical interpretation and the phase nomenclature more precise.
