# Supplementary material for: Crystallographic origin of pressure-induced bond order in the frustrated spinel CuIr2S4
Source: Commun Chem. 2026 Oct 26;9:316. doi: 10.1038/s42004-026-02240-y (PMC13710760; doi:10.1038/s42004-026-02240-y)
Supplement: Supplementary file 2 [file 42004_2026_2240_MOESM2_ESM.pdf]

Supplementary Information

Crystallographic origin of pressure-induced bond order in the frustrated spinel $CuIr_2S_4$

Masatoshi Emi[1,2], Tsubasa Ohashi[1,2], Keita Kojima[1,2,3], Koudai Sugimoto[4,5], Kazuyuki Matsubayashi[6], Kenta Oka[7], Hirokazu Kadobayashi[7], Saori Kawaguchi-Imada[7,8], Daigo Ito[1,2], Taisei Kubo[1,2], Kenta Hashimoto[9], Hitoshi Kawaji[9], Hiroyuki S. Suzuki[3], Shoichi Nagata[10], Naoyuki Katayama[1,2,*]

[1]Department of Applied Physics, Nagoya University, Nagoya 464-8603, Japan
[2]Graduate School of Environmental, Life, Natural Science and Technology, Okayama University, Okayama 700-8530, Japan
[3]The Institute for Solid State Physics, The University of Tokyo, Kashiwa 277-8581, Japan
[4]Department of Physics, Keio University, Kohoku-ku, Yokohama, Kanagawa 223-8522, Japan
[5]Department of Physics, Kitasato University, Minami-ku, Sagamihara, Kanagawa 252-0373, Japan
[6]Department of Engineering Science, The University of Electro-Communications, Chofu, Tokyo 182-8585, Japan
[7]Japan Synchrotron Radiation Research Institute (JASRI), SPring-8, Hyogo 679-5198, Japan
[8]Office of Institutional Advancement and Communications, Kyoto University, Yoshidahonmachi, Sakyo-ku, Kyoto 606-8501, Japan
[9]Materials and Structures Laboratory, Institute of Science Tokyo, 4259 Nagatsuta-cho, Midori-ku, Yokohama 226-8503, Japan
[10]Department of Materials Science and Engineering, Muroran Institute of Technology, Mizumoto-cho, Muroran, Hokkaido 050-8585, Japan

This Supplementary Information provides the constrained Cu/Ir-coordinate refinement and refined structural parameters of Phase II at 300 K and 7.6 GPa, full-atom refinement results for Phase IV at 19.8 and 25.7 GPa, quantitative comparisons between the DFT-relaxed and experimentally refined structures, and additional two-phase fitting results supporting the phase assignments and coexistence regimes discussed in the main text.

Supplementary Note 1. Constrained Rietveld refinement of Phase II

The crystal structure of Phase II at 300 K and 7.6 GPa was analyzed by constrained Rietveld refinement. The ambient-pressure low-temperature single-crystal structure reported by Ohashi *et al*. was used as the initial structural model[1]. Phase II has triclinic $P\bar{1}$ symmetry and contains 28 crystallographically independent sites, all located at general positions. Because the present diamond-anvil-cell powder X-ray diffraction data provide limited sensitivity to the large number of independent structural parameters, particularly the S coordinates, a fully unconstrained all-atom refinement was not stable.

The Cu and Ir atomic coordinates were therefore refined against the high-pressure diffraction data, whereas the S coordinates and atomic displacement parameters were fixed to the values adopted from the ambient-pressure low-temperature structural model. The resulting lattice and atomic parameters are summarized in Supplementary Table 1. The corresponding Rietveld refinement profile is shown in Fig. 2**d** of the main text. This constrained refinement provides an experimentally determined description of the Cu/Ir sublattice in Phase II under pressure, although it is not intended as a fully unconstrained all-atom structure determination.

To separate the effects of lattice compression from those of internal-coordinate relaxation, representative short Ir–Ir distances obtained from the constrained refinement at 300 K and 7.6 GPa were compared with fixed-coordinate reference values at the same pressure. The reference values were calculated by retaining the fractional atomic coordinates of the ambient-pressure low-temperature structure reported by Ohashi *et al*. and substituting the experimentally determined lattice parameters at 7.6 GPa. The uncertainties of these reference distances were evaluated by propagating the standard uncertainties of both the fractional atomic coordinates reported by Ohashi *et al*. and the experimentally determined lattice parameters. The uncertainties of the refined distances were propagated from the refined Ir coordinates and lattice parameters. The results are summarized in Supplementary Table 2. The refined distances are consistent, within their estimated uncertainties, with the fixed-coordinate reference values, supporting the preservation of the characteristic Phase-II short-bond topology under pressure.

The pressure-dependent reference values shown by the orange symbols in Fig. 5**c** of the main text were calculated by retaining the fractional atomic coordinates of the ambient-pressure low-temperature structure reported by Ohashi *et al*. and replacing only the lattice parameters with the experimentally determined values at each pressure. These values represent the geometrical effect of lattice compression without relaxation of the internal atomic coordinates.

Supplementary Table 1. Refined structural parameters of Phase II at 300 K and 7.6 GPa. The ambient-pressure low-temperature single-crystal structure reported by Ohashi *et al*. was used as the initial model. The Cu and Ir atomic coordinates were refined, whereas the S coordinates and atomic displacement parameters were fixed to the values adopted from the ambient-pressure low-temperature structural model. The lattice parameters are a = 11.7515(17) Å, b = 6.88000(68) Å, c = 11.75660(93) Å, α = 91.1617(6)°, β = 108.3500(73)°, and γ = 91.1389(85)°. The refinement reliability factors are R_{wp} = 4.616% and R_p = 2.864%. Because the B_{iso} values were fixed rather than refined, estimated standard uncertainties are not given for these parameters. The rightmost column, d_{diff}, gives the real-space displacement of each refined Cu or Ir site from the corresponding fixed-coordinate reference position calculated using the same 7.6 GPa lattice parameters. The uncertainties of d_{diff} were propagated from the standard uncertainties of the refined atomic coordinates, the reference fractional coordinates, and the experimentally determined lattice parameters at 7.6 GPa. Because the S coordinates were fixed, d_{diff} is not applicable to the S sites.

Atom	Site	Occ.	x/a	y/b	z/c	B_{iso} (Å^2)	d_{diff} (Å)
Cu1	2*i*	1	0.0679(64)	0.2565(95)	0.9408(57)	0.940	0.18(7)
Cu2	2*i*	1	0.4229(59)	0.2608(87)	0.0554(61)	0.940	0.06(6)
Cu3	2*i*	1	0.9444(63)	0.256(12)	0.5553(61)	0.940	0.17(6)
Cu4	2*i*	1	0.5588(62)	0.2792(97)	0.4510(49)	0.940	0.11(8)
Ir1	2*i*	1	0.7344(25)	0.9897(41)	0.2532(22)	0.512	0.07(4)
Ir2	2*i*	1	0.2548(22)	0.0193(36)	0.2622(24)	0.512	0.09(3)
Ir3	2*i*	1	0.2511(25)	0.2252(40)	0.4843(22)	0.512	0.02(3)
Ir4	2*i*	1	0.4877(26)	0.2167(35)	0.7456(24)	0.512	0.04(3)
Ir5	2*i*	1	0.9922(23)	0.2323(36)	0.2502(25)	0.512	0.11(3)
Ir6	2*i*	1	0.7443(24)	0.2380(35)	0.9968(26)	0.512	0.09(3)
Ir7	2*i*	1	0.7406(28)	0.4873(53)	0.2491(25)	0.512	0.11(3)
Ir8	2*i*	1	0.2494(20)	0.5152(37)	0.2493(28)	0.512	0.083(17)
S1	2*i*	1	0.86747	0.46709	0.12839	0.662	–
S2	2*i*	1	0.85877	0.01290	0.13108	0.662	–
S3	2*i*	1	0.37280	0.99905	0.13639	0.662	–
S4	2*i*	1	0.37455	0.54950	0.13202	0.662	–
S5	2*i*	1	0.88929	0.99008	0.63757	0.662	–
S6	2*i*	1	0.87109	0.52859	0.63003	0.662	–
S7	2*i*	1	0.36963	0.45376	0.62526	0.662	–
S8	2*i*	1	0.36710	0.99800	0.60719	0.662	–
S9	2*i*	1	0.36754	0.26750	0.86597	0.662	–
S10	2*i*	1	0.14784	0.24567	0.62621	0.662	–
S11	2*i*	1	0.13093	0.25427	0.14327	0.662	–
S12	2*i*	1	0.36603	0.28028	0.36211	0.662	–
S13	2*i*	1	0.62663	0.23440	0.64058	0.662	–
S14	2*i*	1	0.85688	0.23608	0.86610	0.662	–
S15	2*i*	1	0.85970	0.24807	0.35685	0.662	–
S16	2*i*	1	0.63478	0.24264	0.13121	0.662	–

Supplementary Table 2. Comparison of representative short Ir–Ir distances in Phase II at 7.6 GPa. The fixed-coordinate reference values were calculated using the fractional atomic coordinates of the ambient-pressure low-temperature structure reported by Ohashi *et al*. together with the experimentally determined lattice parameters at 7.6 GPa. The refined values were obtained from the constrained Rietveld refinement at 300 K and 7.6 GPa. The uncertainties of the fixed-coordinate reference values were propagated from the standard uncertainties of the fractional atomic coordinates reported by Ohashi *et al.*[1] and those of the experimentally determined lattice parameters at 7.6 GPa. The uncertainties of the refined distances were propagated from the refined Ir coordinates and lattice parameters.

Ir–Ir pair	Fixed-coordinate reference at 7.6 GPa (Å)	Constrained refinement at 7.6 GPa (Å)
Ir1–Ir4, short bond 1	2.9198(6)	2.96(5)
Ir2–Ir3, short bond 2	2.9282(4)	2.94(4)

Supplementary Note 2. Full-atom structure refinement of Phase IV

In this section, we summarize the structural models used for Phase IV and the results of the full-atom Rietveld refinements for the single-phase datasets collected at 19.8 and 25.7 GPa. Supplementary Table 3 lists the DFT-relaxed structural parameters used as the initial model, while Supplementary Tables 4 and 5 summarize the refined atomic parameters obtained from the 19.8 and 25.7 GPa datasets, respectively. These results provide the supplementary basis for the Phase IV structure determination described in the main text.

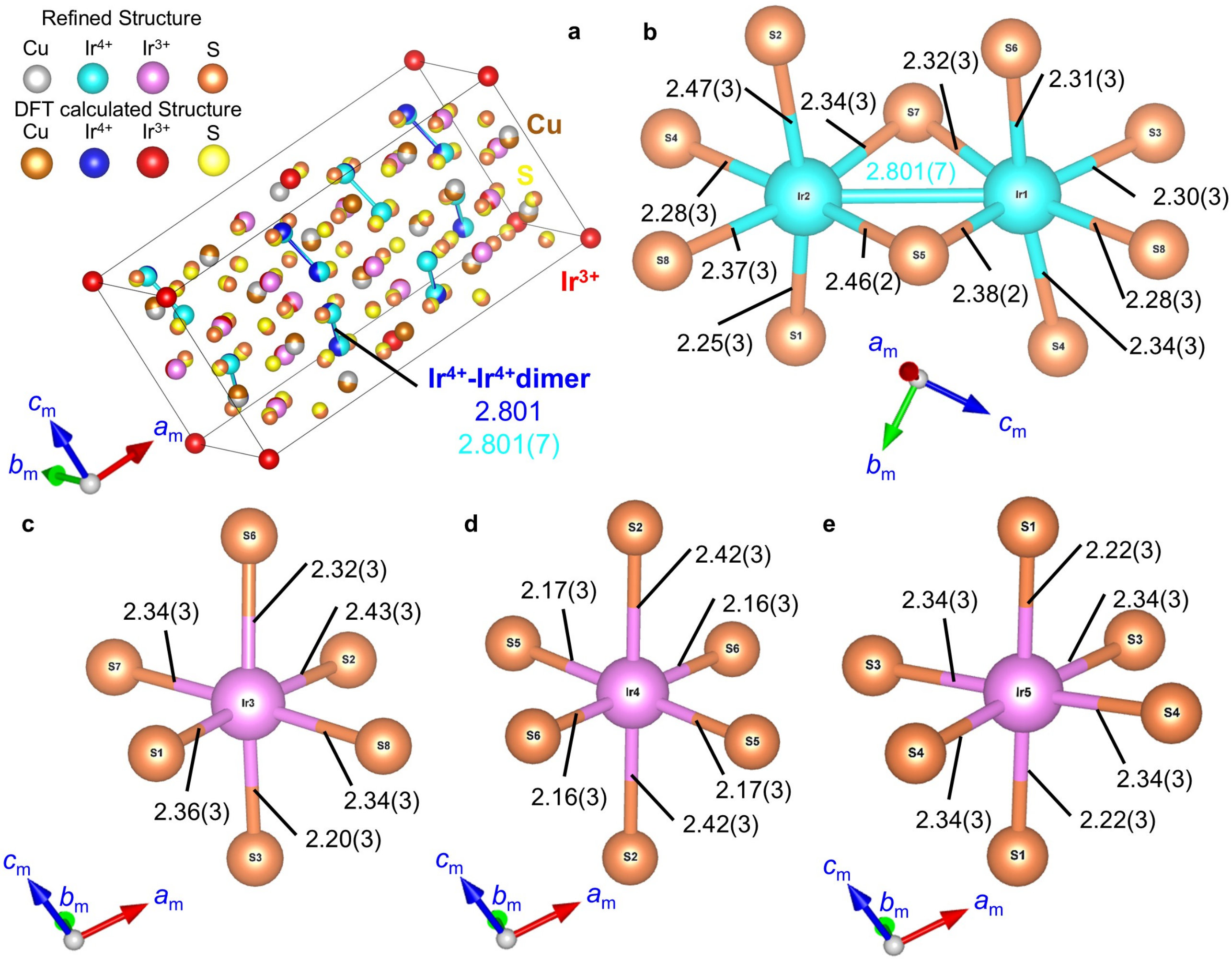

Supplementary Figure 1. Comparison of the DFT-relaxed and experimentally refined Phase-IV structures. **a,** Comparison between the DFT-relaxed Phase-IV structure and the structure obtained by full-atom Rietveld refinement of the diffraction dataset collected at 300 K and 19.8 GPa. The DFT-relaxed structure was used as the initial model for the refinement. For this comparison, both structures were expressed using the experimentally refined lattice parameters at 19.8 GPa, allowing a direct comparison of the corresponding atomic positions. The Ir^{3+}/Ir^{4+} labels are nominal assignments used solely to indicate the short-bond topology within an idealized ionic picture and do not imply complete integer charge separation. The colors assigned to the constituent elements and to the DFT-relaxed and refined structures are indicated in the figure. **b–e,** Representative Ir–S bond lengths derived from the experimentally refined structure. Panel **b** shows the Ir_2S_{10} cluster containing the short Ir–Ir bond, whereas panels **c–e** show the IrS_6 octahedra surrounding the remaining crystallographically independent Ir sites. The atomic-site labels correspond to those listed in Supplementary Table 4.

Supplementary Table 3. Structural parameters of the DFT-relaxed Phase-IV model in space group $C2/c$. A diffraction-informed Phase-IV structural model constructed from the data collected at 19.8 GPa was used as the starting structure for DFT relaxation. Both the lattice parameters and internal atomic coordinates were optimized. The optimized lattice parameters are $a = 22.5988$ Å, $b = 6.7196$ Å, $c = 11.1564$ Å, and $\beta = 99.4962°$. The fractional atomic coordinates of the DFT-relaxed structure were subsequently expressed using the experimentally determined lattice parameters at 19.8 GPa and used as the initial model for the full-atom Rietveld refinement of the diffraction dataset collected at 300 K and 19.8 GPa. All crystallographic sites were fully occupied. Atomic displacement parameters are not listed because this table presents a DFT-relaxed structure rather than parameters refined against diffraction data.

Atom	Site	Occ.	x/a	y/b	z/c
Cu1	8f	1	0.27652	0.27054	0.83993
Cu2	8f	1	0.03134	0.21766	0.10196
Ir1	8f	1	0.12450	0.28767	0.61299
Ir2	8f	1	0.87575	0.47699	0.11004
Ir3	8f	1	0.87680	0.02155	0.62541
Ir4	4d	1	0.25	0.25	0.5
Ir5	4e	1	0	0.71439	0.25
S1	8f	1	0.43667	0.23219	0.06628
S2	8f	1	0.18314	0.23196	0.32168
S3	8f	1	0.43602	0.44437	0.31078
S4	8f	1	0.43396	0.01632	0.80392
S5	8f	1	0.69552	0.00445	0.56196
S6	8f	1	0.68359	0.47355	0.06013
S7	8f	1	0.56381	0.27843	-0.06995
S8	8f	1	0.82122	0.27667	0.69129

Supplementary Table 4. Refined structural parameters obtained from the full-atom Rietveld refinement of Phase IV at 300 K and 19.8 GPa, corresponding to the result shown in Fig. 3f of the main text. The fractional atomic coordinates of the DFT-relaxed Phase-IV model listed in Supplementary Table 3 were expressed using the experimentally determined lattice parameters at 19.8 GPa and used as the initial structural model. The refinement reliability factors were $R_{\mathrm{wp}} = 2.428\%$ and $R_{\mathrm{p}} = 1.755\%$. The refined lattice parameters are a = 22.68668(36) Å, b = 6.74483(11) Å, c = 11.18487(23) Å, and β = 99.6629(16)°. The site occupancies were fixed to unity. The atomic displacement parameters were constrained to be identical within each element species, except for Ir, for which the sites involved in dimer formation and those not involved in dimer formation were refined as two separate groups. All crystallographic sites belonging to the same constrained group therefore share the same refined B_{iso} value and estimated standard uncertainty. The rightmost column, d_{diff}, gives the real-space displacement between each refined atomic position and the corresponding position obtained from the fractional coordinates of the DFT-relaxed model listed in Supplementary Table 3. For this comparison, the DFT-relaxed fractional coordinates were expressed using the experimentally refined lattice parameters at 19.8 GPa. The uncertainties of d_{diff} were propagated from the refined atomic-coordinate and lattice-parameter uncertainties, with the DFT-relaxed fractional coordinates treated as fixed. Across the crystallographically independent sites, the maximum and root-mean-square positional differences between the DFT-relaxed and refined structures are 0.214 and 0.120 Å, respectively. The root-mean-square value was calculated with equal weighting over the crystallographically independent sites. For the Ir sublattice alone, the corresponding maximum and root-mean-square positional differences are 0.033 and 0.025 Å, respectively.

Atom	Site	Occ.	x/a	y/b	z/c	B_{iso} (Å^2)	d_{diff} (Å)
Cu1	8f	1	0.28127(48)	0.2678(15)	0.85062(96)	1.066(87)	0.149(10)
Cu2	8f	1	0.02679(43)	0.2116(13)	0.08458(93)	1.066(87)	0.214(10)
Ir1	8f	1	0.12329(20)	0.28718(39)	0.61340(44)	0.670(44)	0.027(5)
Ir2	8f	1	0.87601(17)	0.48080(61)	0.10785(37)	0.670(44)	0.033(5)
Ir3	8f	1	0.87793(18)	0.02327(63)	0.62599(49)	0.602(41)	0.032(5)
Ir4	4d	1	0.25	0.25	0.5	0.602(41)	0
Ir5	4e	1	0	0.71788(69)	0.25	0.602(41)	0.018(5)
S1	8f	1	0.43959(99)	0.2209(32)	0.0725(24)	0.247(84)	0.12(3)
S2	8f	1	0.18452(99)	0.2313(33)	0.3063(24)	0.247(84)	0.18(3)
S3	8f	1	0.4324(12)	0.4478(33)	0.3072(26)	0.247(84)	0.09(3)
S4	8f	1	0.43242(94)	0.0194(28)	0.7973(28)	0.247(84)	0.08(3)
S5	8f	1	0.70321(72)	-0.0087(32)	0.5717(21)	0.247(84)	0.208(18)
S6	8f	1	0.68545(85)	0.4512(39)	0.0539(26)	0.247(84)	0.17(3)
S7	8f	1	0.56024(93)	0.2747(32)	-0.0690(24)	0.247(84)	0.09(3)
S8	8f	1	0.82156(89)	0.2755(27)	0.6967(21)	0.247(84)	0.06(3)

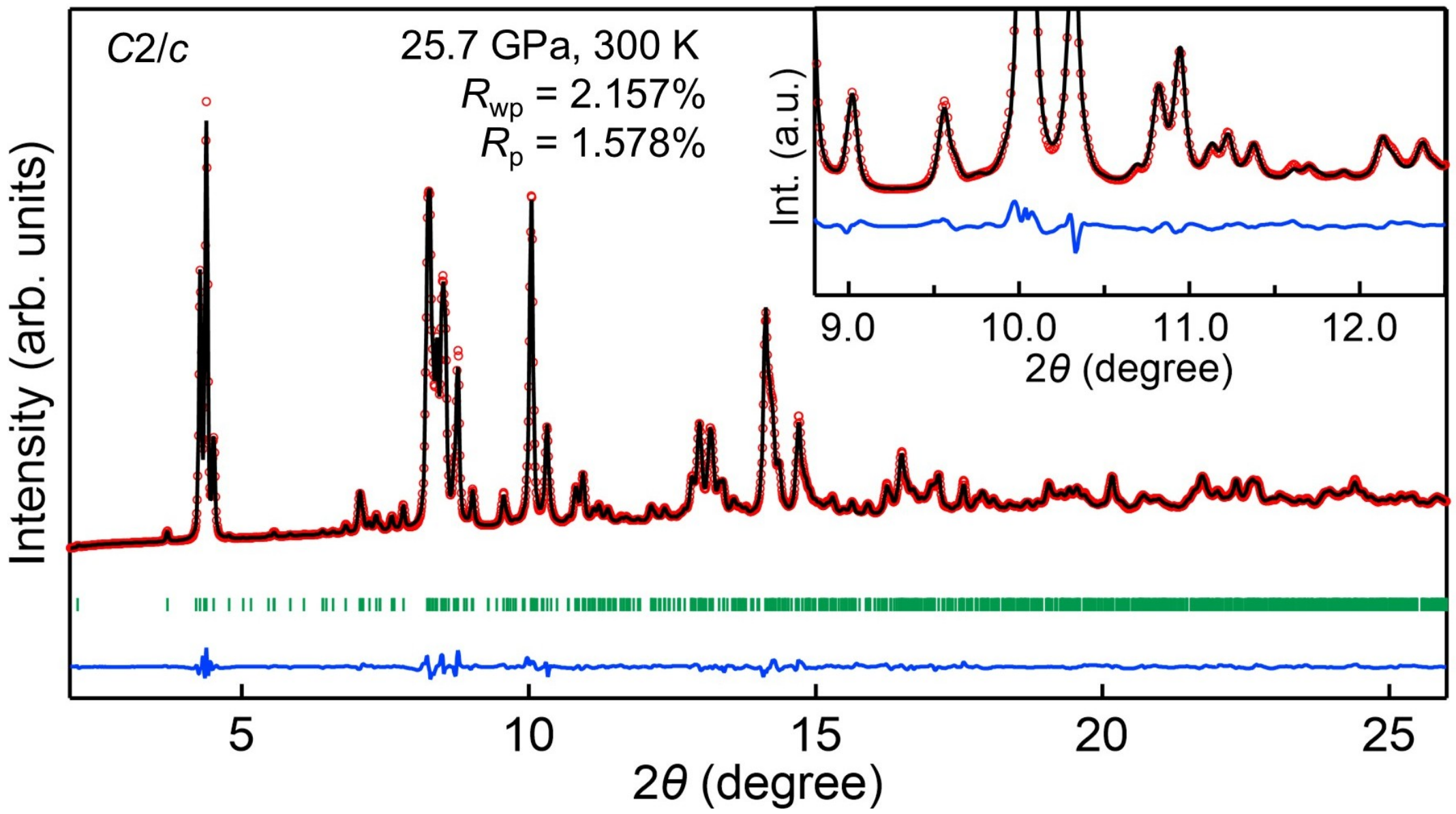

Supplementary Figure 2. Final Rietveld refinement for the single-phase Phase-IV dataset collected at 300 K and 25.7 GPa. The fractional atomic coordinates of the DFT-relaxed model listed in Supplementary Table 3 were expressed using the experimentally determined lattice parameters at 25.7 GPa and used as the initial structural model. The atomic coordinates of all constituent elements were refined in space group *C*2/*c*. The inset displays an enlarged view of the 8.8°–12.5° region. Red circles represent the observed intensities, the black line the calculated profile, the blue line the difference curve, and the green ticks the Bragg reflection positions.

Supplementary Table 5. Refined structural parameters obtained from the full-atom Rietveld refinement of Phase IV at 300 K and 25.7 GPa. The refinement was performed in space group $C2/c$. The fractional atomic coordinates of the DFT-relaxed Phase-IV model listed in Supplementary Table 3 were expressed using the experimentally determined lattice parameters at 25.7 GPa and used as the initial structural model. The atomic coordinates of all constituent elements were subsequently refined against the diffraction data. The refinement reliability factors are R_{wp} = 2.157% and R_p = 1.578%. The refined lattice parameters are a = 22.51246(37) Å, b = 6.69261(11) Å, c = 11.06525(28) Å, and β = 99.6896(18)°. The site occupancies were fixed to unity. The atomic displacement parameters were constrained to be identical within each element species, except for Ir, for which the sites involved in dimer formation and those not involved in dimer formation were refined as two separate groups. All crystallographic sites belonging to the same constrained group therefore share the same refined B_{iso} value and estimated standard uncertainty. The rightmost column, d_{diff}, gives the real-space displacement between each refined atomic position and the corresponding position obtained from the fractional coordinates of the DFT-relaxed model listed in Supplementary Table 3, expressed using the refined lattice parameters at 25.7 GPa. The uncertainties of d_{diff} were propagated from the refined atomic-coordinate and lattice-parameter uncertainties, with the DFT-relaxed fractional coordinates treated as fixed. This refinement provides an independent consistency check of the Phase-IV structural model determined from the 19.8 GPa dataset.

Atom	Site	Occ.	x/a	y/b	z/c	B_{iso} (Å^2)	d_{diff} (Å)
Cu1	8f	1	0.27570(55)	0.2806(13)	0.8425(12)	1.547(72)	0.076(10)
Cu2	8f	1	0.02909(50)	0.2207(12)	0.0949(11)	1.547(72)	0.088(11)
Ir1	8f	1	0.12372(20)	0.28997(36)	0.61272(45)	0.524(41)	0.023(4)
Ir2	8f	1	0.87518(17)	0.47532(51)	0.10809(40)	0.524(41)	0.026(4)
Ir3	8f	1	0.87663(21)	0.02189(58)	0.62592(55)	0.679(39)	0.008(6)
Ir4	4d	1	0.25	0.25	0.5	0.679(39)	0
Ir5	4e	1	0	0.71600(63)	0.25	0.679(39)	0.011(5)
S1	8f	1	0.4378(11)	0.2246(29)	0.0658(24)	0.118(82)	0.06(3)
S2	8f	1	0.1867(11)	0.2243(29)	0.3116(25)	0.118(82)	0.16(3)
S3	8f	1	0.44474(84)	0.4406(24)	0.3278(21)	0.118(82)	0.249(19)
S4	8f	1	0.43022(83)	-0.0003(25)	0.7940(28)	0.118(82)	0.17(3)
S5	8f	1	0.69664(80)	0.0298(31)	0.5638(21)	0.118(82)	0.17(3)
S6	8f	1	0.67737(85)	0.4714(29)	0.0333(24)	0.118(82)	0.31(3)
S7	8f	1	0.56150(79)	0.2768(30)	-0.0767(19)	0.118(82)	0.085(18)
S8	8f	1	0.82362(89)	0.2668(27)	0.7083(21)	0.118(82)	0.20(3)

The experimentally refined full-atom Phase-IV structure at 19.8 GPa listed in Supplementary Table 4 has been deposited with the Cambridge Crystallographic Data Centre under deposition number CCDC 2551065. The DFT-relaxed initial model is fully specified in Supplementary Table 3, whereas the 25.7 GPa refinement in Supplementary Table 5 is provided as an independent consistency check.

Supplementary Note 3. Additional analysis results supporting the content of the main text

Here, we present several additional analytical results that support the findings of this paper.

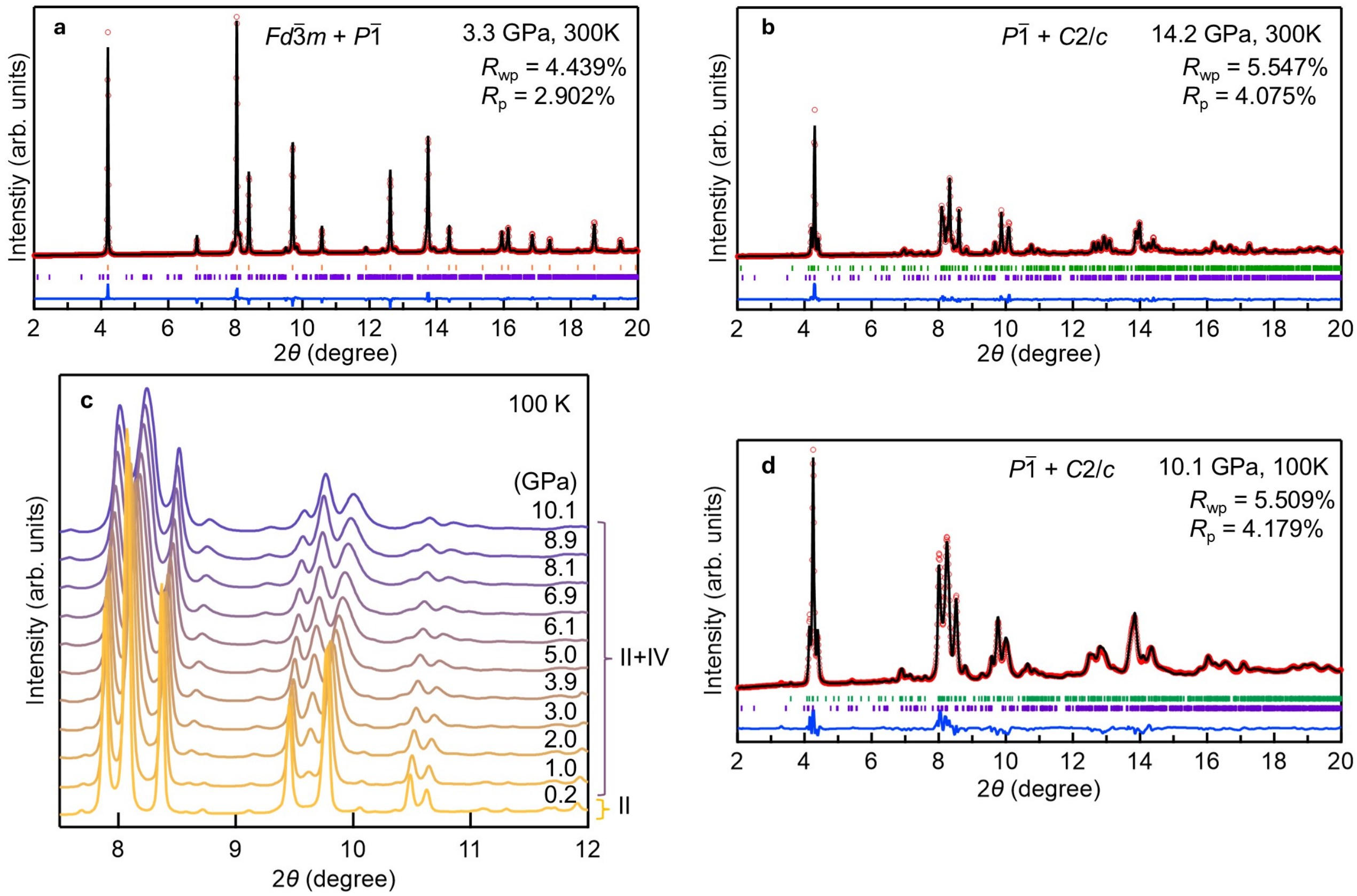

Supplementary Figure 3. Additional two-phase fits and low-temperature diffraction patterns. **a**, Two-phase fit for the I+II coexistence region (Phase I: $Fd\bar{3}m$; Phase II: $P\bar{1}$) at 3.3 GPa and 300 K. The molar ratio is Phase I : Phase II = 0.858(3) : 0.142(3). Orange ticks indicate Phase I and purple ticks indicate Phase II. **b**, Two-phase fit for the Phase-II+IV coexistence region at 14.2 GPa and 300 K. The molar ratio is Phase II : Phase IV = 0.303(3) : 0.697(2). Green ticks indicate Phase II and purple ticks indicate Phase IV. **c**, Pressure evolution of the diffraction patterns at 100 K. Compared with the 300 K data, the coexistence regime (Phase II+IV) shifts to lower pressure, consistent with a dome-like stability region of the high-pressure phase as depicted in Fig. 1 of the main text. **d**, Two-phase fit for the Phase-II+IV coexistence region at 10.1 GPa and 100 K. The molar ratio is Phase II : Phase IV = 0.327(3) : 0.673(3). Green ticks indicate Phase II and purple ticks indicate Phase IV. In panels **a**, **b**, and **d**, red circles represent the observed intensities, black lines the calculated profiles, and blue lines the difference curves.

Supplementary References

1. Ohashi, T. *et al.* Reexamination of the charge-ordered dimer pattern in the spinel compound $CuIr_2S_4$ using single-crystal synchrotron x-ray diffraction. *Phys. Rev. B* **111**, 224114 (2025).
